# “I Just Can’t Take It Anymore”: How Specific Work Characteristics Impact Younger Versus Older Nurses’ Health, Satisfaction, and Commitment

**DOI:** 10.3389/fpsyg.2020.00762

**Published:** 2020-05-27

**Authors:** Beatrice I. J. M. Van der Heijden, Inge Houkes, Anja Van den Broeck, Katarzyna Czabanowska

**Affiliations:** ^1^Institute for Management Research, Radboud University, Nijmegen, Netherlands; ^2^School of Management, Open University of the Netherlands, Heerlen, Netherlands; ^3^Department of Marketing, Innovation and Organisation, Ghent University, Ghent, Belgium; ^4^Business School of Hubei University, Hubei University, Wuhan, China; ^5^Kingston Business School, Kingston University, London, United Kingdom; ^6^Social Medicine, Care and Public Health Research Institute (CAPHRI), Maastricht University, Maastricht, Netherlands; ^7^Department of Work and Organization Studies, KU Leuven, Leuven, Belgium; ^8^Optentia, North West University, Vanderbijlpark, South Africa; ^9^Department of International Health and Care, Public Health Research Institute, Maastricht University, Maastricht, Netherlands; ^10^Institute of Public Health, Faculty of Health Sciences, Jagiellonian University, Krakow, Poland

**Keywords:** nurses, labor relations, work content, conditions of employment, burnout, job satisfaction, institutional affective commitment, age

## Abstract

Given the increasing shortage of active nurses in industrialized countries throughout the world, it is of utmost importance to protect their health, satisfaction, and commitment so that they can continue working in their healthcare institution. Building upon the proposed pattern of specific relationships developed by Houkes et al. (2003), we investigated a model of relationships among working conditions (quantitative, emotional, and physical demands), labor relations (quality of interpersonal relations and psychological support), work content (meaning of work, influence at work), and employment conditions (opportunities for development) on the one hand, and health, job satisfaction, and institutional affective commitment on the other hand, for younger versus older nurses. We used data of 3,399 nurses from the Netherlands and 3,636 nurses from Poland from the larger European Nurses’ Early Exit Study (NEXT) and performed longitudinal structural equation modeling (SEM) and multi-group analyses. The results showed that the proposed pattern of relationships generally holds, but that the nurses’ level of commitment is more determined by meaning of work than by opportunities for development and that psychological support is associated with job satisfaction (and not only with burnout as hypothesized, in both the Netherlands and Poland). Comparing younger (<40 years) versus older (≥40 years) nurses, we found ample support for differences in the proposed model relationships across age category, some being in line with and some being contradictory to our expectations. We argue that a non-normative, tailor-made approach to aging at work might help us to protect the nurses’ career sustainability across the life span. This study provides evidence-based practical recommendations on how to enhance the health, job satisfaction, and commitment of nurses throughout their working life.

## Introduction

Demographic changes such as aging and dejuvenization have increased the need for care in Europe (Wang and Shi, 2014). At the same time, Europe faces a considerable shortage of nurses (Chan et al., 2013) due to the “baby boomer” generation approaching (early) retirement and the high level of premature professional turnover (Buchan et al., 2015). This is not expected to improve in the short run (Chan et al., 2013).

Nursing is a highly demanding profession which entails high risks for health problems (Aiken et al., 2001; Mark and Smith, 2012), limiting nurses’ work ability (Ilmarinen, 1999) and employability (career potential) (Fugate et al., 2004; Van der Heijden et al., 2009), and herewith their capacity to work until official retirement age. In addition, many nurses leave the nursing profession, which they consider unattractive and extremely burdensome, due to the nature of the work, no sense of purpose of their own work, and negative interpersonal relations, as well as a disconnection with the profession and the institution, lack of job satisfaction, burnout, and low assessment of their own health (Aiken et al., 2012).

Nurses’ health, job satisfaction (Lu et al., 2019), and organizational commitment (Brunetto et al., 2013) are among the key factors that predict whether they are able to stay active in the labor market and to prevent premature leave (Hasselhorn et al., 2003). Based on the literature on work characteristics (Parker et al., 2017a) and highly influential models in the scholarly domain of aging (Ilmarinen, 2007; Brady et al., 2019), work characteristics can be seen as of essential importance to maintain or even improve employees’ health, job satisfaction, and organizational commitment and – therefore – their work ability, especially of older workers. It is therefore of utmost importance to understand the impact of work characteristics on nurses’ health, satisfaction, and commitment and age-related differences therein.

As a part of the European Nurses’ Early Exit Study (NEXT) research project,^1^ this study aims to increase our understanding of how work characteristics subdivided into four clear categories (in line with Houkes et al., 2001) – (1) working conditions, (2) labor relations, (3) work content, and (4) conditions of employment (see also Kompier and Marcelissen, 1990; Kompier and Di Martino, 1995) – impact over time on nurses’ health (i.e., burnout, disability), job satisfaction (as a motivational variable), and institutional affective commitment (as a career-related variable). Building on the Selection, Optimization, and Compensation Theory (SOC) and the Socio-emotional Selectivity Theory (SST) – being two complementary perspectives on aging at work – we moreover investigate whether this pattern of relationships is different for younger (<40 years) versus older (≥40 years) nurses [see Finkelstein and Farrell, 2007, p. 100, on the Age Discrimination in Employment Act (ADEA); see also Boerlijst et al., 1998; Taylor and Walker, 1998; Van der Heijden et al., 2009, for justification for this dichotomy in research that has been conducted in Europe]. Although age is often included as a covariate or confounder in research studying the associations between work characteristics and outcomes, more empirical work is needed to study if and how age affects relationships between model variables (De Lange et al., 2010).

Considering the recommendations by Lave and March (1990) and Kristensen (1996), and in line with Houkes et al. (2001), we will test our proposed research model in two different countries. This will help us to gain more insight into the robustness and generalizability of our study and adds to the paucity of comparative career research (Thomas and Inkson, 2007). In particular, we will focus on the Netherlands and Poland, which both experience a severe shortage of nurses due to the increase in healthcare needs of the aging society, the rising incidence of chronic diseases and disabilities, retiring older generations of nurses, the emigration of staff, and changes in the educational system (Zgliczyński et al., 2016; Taskforce Healthcare, 2017). The problems related to the shortages of the workforce are thus similar in both countries, however, in the literature some overlapping and divergent experiences have been reported among nurses in the Netherlands and Poland, which makes it particularly relevant to examine whether one theoretical model can explain these experiences. Levels of emotional exhaustion, for example, are lower in the Netherlands than in Poland (Huisman-de Waal et al., 2019; Kózka et al., 2019). Eight out of 10 Dutch nurses are proud to work in their healthcare setting but would appreciate more managerial support and recognition. Polish nurses’ desire to leave is related to organizational and socio-economic factors, such as bad working conditions, interpersonal conflicts, wrong management system, low status of work in social hierarchy, shortage of human resources, job insecurity, competition, lack of trust, and low salaries in relation to their efforts. Given the similarities and differences between both countries, we argue that they are a good basis for our empirical research.

The outline given above stresses that, apart from adding to the theory development on the impact of work characteristics on nurses’ health, job satisfaction, and organizational commitment, this study also has practical merits in providing healthcare managers in the Netherlands and Poland with evidence-based advice on how to fine-tune work characteristics for nurses across the life span.

## Theoretical Framework

### Work Characteristics as Predictors of Health, Job Satisfaction, and Commitment

Work design, which is defined as the content and organization of one’s work tasks, activities, relationships, and responsibilities (Parker, 2014, p. 662), has implications for important employee and organizational outcomes, including health and well-being, motivation, innovation, and performance. In their seminal work, Houkes et al. (2001) aimed to contribute to the work design literature by improving and refining exemplary work design models, such as the model for Work, Stress, and Health (Kompier and Marcelissen, 1990; Kompier and Di Martino, 1995), the Demand–Control–Support model (DCS model; Johnson and Hall, 1988; Karasek and Theorell, 1990), and the Effort–Reward Imbalance model (ERI model; Siegrist, 1996). Notwithstanding the contribution of these models to the theorizing in the field of work psychology and their added value for the design of employee jobs, Houkes et al. (2001) argued that the work design literature was in need of more refined models to better understand specific patterns of relationships between various work characteristics and outcome variables and to suggest evidence-based interventions at the workplace.

To arrive at more specific and testable hypotheses, Houkes et al. (2001) studied how various characteristics of the working conditions, labor relations, work content, as well as conditions of employment yield specific relationships with outcomes such as exhaustion, as an indicator of employees’ health and well-being; job satisfaction, being a motivational outcome; and turnover intentions, which can be considered a career outcome. They argued and found that emotional exhaustion was most strongly predicted by workload, which is indicative of the conditions under which work needs to be done, and social support, referring to an aspect of the social and labor relations at work. In line with Hobfoll (1989); Houkes et al. (2001) posited that this is the case because workload threatens people’s abilities to maintain and obtain resources, which in turns triggers emotional exhaustion. Social support, in contrast, is considered a valuable resource, as it brings direct instrumental help, feedback, information, or emotional support (House, 1981), and is helpful in buffering the demanding characteristics of one’s work (Bakker et al., 2005). The availability of social support may therefore increase employees’ pool of resources and contribute to their health and prevent emotional exhaustion.

Intrinsic motivation was primary predicted by work content variables such as feedback, autonomy, and skill use. These factors are enjoyable aspects of the job itself and speak to the interest of employees, and, following Hackman and Oldham (1976), these factors have great motivating potential. Finally, Houkes et al. (2001) argued and found that turnover was most strongly predicted by employment conditions such as unmet expectations regarding one’s salary, job security, and position. The lack of good employment conditions frustrates employees’ growth needs, and as a result, it is likely that employees are pushed to look for growth opportunities elsewhere.

Several studies have provided support for the applicability of the model of Houkes et al. (2001) in the nursing sector. More specifically, these studies showed that job control was consistently a stronger predictor of motivational outcomes such as job satisfaction, while job demands consistently were the strongest predictors of emotional exhaustion (primary indicator of burnout) (Tummers et al., 2002; Janssen et al., 2004). We aim to build on the model of Houkes et al. (2001) by studying the specific longitudinal effects of several aspects of nurses’ working conditions (quantitative, emotional, and physical demands), labor relations (quality of interpersonal relations and psychological support), work content (meaning of work and influence at work), and conditions of employment (opportunities for development) on nurses’ burnout and disability as health indicators, job satisfaction as a motivational outcome, and institutional affective commitment as a career-related outcome. Earlier empirical work in the nursing sector has already shown that these variables matter. For example, meta-analytic findings indicate that nurses who are confronted with working conditions such as high workload and a lack of social relations are more prone to burnout (Li et al., 2018). Other working conditions such as quantitative, emotional, and physical demands are furthermore an important risk factor for nurses’ well-being, both in their personal life and at the workplace (see, for instance, Van der Heijden et al., 2008), while social support of one’s direct supervisor and near colleagues are among the most important factors to maintain health and well-being when nurses face challenging work demands (Estryn-Behar et al., 2007; Van der Heijden et al., 2017).

Furthermore, an overview of the literature indicates that work content variables such as meaningfulness and control are among the most important work aspects that contribute to nurses’ motivation for the job and are associated with nurses’ job satisfaction (Toode et al., 2011; Lu et al., 2019). Finally, nurses’ turnover intent increases when employment conditions, such as opportunities for growth and development as well as pay and salary, are unsatisfactory (Flinkman et al., 2010; Halter et al., 2017). Perceived organizational support for further development (Tansky and Cohen, 2001) and career growth (Weng et al., 2010; Weng and McElroy, 2012) also appear to be associated with organizational commitment, being one of the outcome indicators in our research model.

**FIGURE 1:**
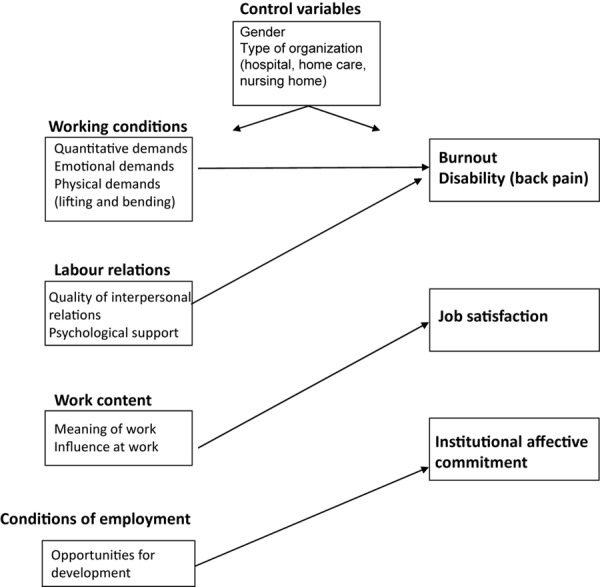
Research model: hypothesized pattern of relationships.

Although these studies convincingly show that working conditions, labor relations, work content, and employment conditions matter for nurses’ health, motivation and career outcomes, they did not systematically test the relative importance of these categories of work characteristics in predicting these outcomes as proposed by Houkes et al. (2001). For example, the reviews by Lu et al. (2019) and by Toode et al. (2011) also indicate that demanding working conditions are associated with job satisfaction, being our motivational outcome, and that social relations contribute to nurses’ motivation as well, yet the relative contributions of working conditions and labor relations vis-à-vis work content and conditions of employment in the prediction of outcomes were not examined. In order to bring more specificity in the way we theorize and study the impact of work characteristics among nurses, and to allow for specific interventions, we tie in with the model of Houkes et al. (2001) and formulate the following hypotheses (see Figure 1):

*Hypothesis 1:* Nurses’ health, in terms of their burnout and disability, is primarily predicted by working conditions (quantitative, emotional, and physical demands) and labor relations (quality of interpersonal relations and psychological support). More specifically, burnout is mainly predicted by quantitative demands, emotional demands, quality of interpersonal relations, and psychological support, and disability is mainly predicted by physical demands.

*Hypothesis 2*: Nurses’ job satisfaction is primarily predicted by work content (meaning of work and influence at work).

*Hypothesis 3*: Nurses’ institutional affective commitment is primarily predicted by conditions of employment (opportunities for development).

Please note: Our current selection of indicators of the four areas of work characteristics in the NEXT data set expands upon the theoretical model and the selection of variables of Houkes et al. (2001) and the initial work of Kompier and colleagues (Kompier and Marcelissen, 1990; Kompier and Di Martino, 1995). Working conditions refer to the amount of work, the physical conditions, and the safety issues related to work. In line with this definition, we included three different types of work demands (i.e., quantitative, emotional, and physical demands), thereby expanding the work of Houkes et al. (2001), who focused on quantitative demands only. Labor relations refer to the (formal and informal) relationships and interactions employers and employees have with each other and the amount of support these relationships provide (Kompier and Marcelissen, 1990; Houkes et al., 2001, 2003). Although social support was not an available measure in the NEXT data set, we were able to include quality of interpersonal relations and psychological support, thereby stretching the operationalization of this construct in the work by Houkes et al. (2001). Work content refers to content and characteristics of the tasks to be done (e.g., Kompier and Marcelissen, 1990). For this category of work characteristics, we have included meaning of work and influence at work, which resembles the operationalization of Houkes et al. (2001) (that is, motivating potential, consisting of autonomy and task significance). Conditions of employment refer to the agreements that are being made between employee and the organization, and concerns aspects such as salary and career and growth opportunities (Kompier and Marcelissen, 1990). Houkes et al. (2001) used the more negative indicator “unmet career expectations” including expectations around salary, position, and job security. We focus on opportunities for development as a good alternative indicator for conditions of employment in the contemporary labor market, as it focuses on one’s intrinsic growth opportunities in the job.

### The Impact of Nurses’ Age

Because the workforce is aging, researchers are increasingly examining how older employees – compared to their younger colleagues – experience their work and work setting. While considerable attention has been devoted to whether maintenance- (e.g., participation) and growth-related (e.g., training and development) HR practices have different implications for workers differing in age (e.g., Kooij et al., 2010), the job design literature has been relatively silent about the potential differences in the impact of work characteristics on older and younger employees’ health and well-being, on their motivation (see Schreurs et al., 2012, for a cross-sectional paper example), and on (other) career-related outcomes.

To formulate specific hypotheses, we borrow from life span development theories, in particular, the SOC (Baltes et al., 1999) and SST (Carstensen, 1995), in arguing that older nurses face the challenges of loss, growth, and change over time (Kanfer and Ackerman, 2004). Older workers, for example, experience a decline in physical capacities and fluid intelligence yet an incline in crystallized intelligence and experiences. Moreover, the salience of particular life goals fluctuates depending on one’s life cycle, with some goals gaining more importance and others becoming less valuable when employees grow older. All this, in turn, may influence how the various aspects of work conditions, labor relations, work content, and conditions of employment influence employee health, motivational, and career-related outcomes.

First, because older employees are confronted with several losses, they may become more sensitive to demanding situations. We argue that this may be the case because such situations may appeal to the impaired skills, abilities, or energy of older workers or require them to mobilize all remaining energy and other resources to overcome these demands (Hobfoll, 1989; Hobfoll et al., 2018). Given that older employees may lack the relevant compensatory resources, they would rather use other strategies, such as preventing such demanding situations from happening or disengaging from situations that pose unattainable goals (Moghimi et al., 2017). Within the work context, however, nurses are unlikely to be able to avoid all demanding working conditions. Therefore, we expect that the health-impairing impact (Bakker et al., 2005) of demanding working conditions, such as quantitative, emotional, and physical demands, may be stronger for older than for younger workers. More specifically, we assume:

*Hypothesis 4*: Age impacts the relationship between working conditions and health such that the positive relationship between quantitative and emotional demands and nurses’ burnout and the positive relationship between physical demands and nurses’ disability are stronger for older compared to younger workers.

Second, because of the experience of loss, older employees are also likely to become more sensitive to resourceful situations, which are beneficial in and of themselves but are especially valuable because they help older employees to deal with the wide range of demands they are confronted with (Hobfoll, 1989; Hobfoll et al., 2018). Not all resources may, however, be experienced as equally beneficial. Following the SST (Carstensen, 1995), with increasing age, one’s time frame changes, causing older employees to value different things than younger employees. While younger nurses may perceive time as more “open-ended” (Carstensen, 1995; Kooij et al., 2018; Dordoni et al., 2019), older nurses’ future time perspective is more limited and framed in terms of the “remaining time.” Younger employees are therefore more likely to focus on all possibilities lying ahead and value keeping all options open. This translates, for example, into attaching high importance to extrinsic rewards, such as pay and promotion, as these help to achieve other things in life. In addition, younger workers also highly value opportunities for development. Even when such opportunities do not serve immediate payoff, they might come in handy somewhere in the future. This line of reasoning also applies to social relations. Younger employees have a wish to expand their social network, as new people may help them to gain new knowledge and information and may be helpful to reach future goals.

Older employees, in contrast, are more oriented toward making the remaining time count. They therefore have a stronger focus on the present and prioritize emotional well-being over growth and learning. Older workers therefore highly value intrinsic qualities of a job, such as the work content, rather than opportunities for growth and development, and they attach more importance to the quality of relationships in comparison with their quantity. Rather than having more relationships, they deepen their existing ones and value the support they get from these. Therefore, we argue that for the older nurses, the impact of the quality of their interpersonal relationships with various stakeholders, ranging from nursing management to colleagues, and the quality of the psychological support (e.g., in terms of their satisfaction with the support provided at work) are especially important for their emotional well-being (i.e., prevention of burnout). Given that we expect labor relations to be one of the primary predictors of health, work content to be the primary predictor of job satisfaction, and opportunities for development to be the primary predictor of organizational commitment, we therefore formulate:

*Hypothesis 5:* Age impacts the relationship between labor relations and health such that the positive relationship between the quality of interpersonal relations and psychological support on the one hand and nurses’ burnout on the other hand is stronger for older compared to younger workers.

*Hypothesis 6:* Age impacts the relationship between work content and job satisfaction such that the positive relationship between meaning of work and influence at work with nurses’ job satisfaction is stronger for older compared to younger workers.

*Hypothesis 7:* Age impacts the relationship between conditions of employment and institutional affective commitment such that the positive relationship between opportunities for development and institutional affective commitment is weaker for older workers.

## Methodology

### Design, Participants, and Procedure

This study comprises an incomplete two-wave panel design (Zapf et al., 1996) utilizing the Dutch and the Polish part of the database of a large European survey study on nurses’ reasons, circumstances, and consequences surrounding premature departure from the nursing profession (NEXT). The NEXT study has been approved by the ethical committee of the University of Wuppertal in Germany. Stratified sampling has been used in order to, as far as possible, reflect the national distribution of nurses working in the Netherlands and in Poland in three different types of institutions (hospitals, nursing homes, and home care institutions) and to cover the different regions in the two countries in a representative way. In particular, the researchers have tried their best to ensure proportionate ratios regarding employment figures, gender distribution, age structure, and working hours across the distinguished types of institutions, while at the same time incorporating the geographical spread across the specific regions in the Netherlands and Poland. To reach this goal, a thorough analysis of the healthcare industry across the two countries, and the population of nurses working in it, was conducted in order to make sure that the study sample would be representative of the participating country’s industry breakdown as regards the distinguished criteria.

The longitudinal design of the study (1-year time lag) comprised a baseline questionnaire and a follow-up questionnaire covering aspects of nurses’ working and private lives, which were sent to a total of 9,309 Dutch and 7,091 Polish nurses at baseline measurement. These two samples covered all nurse qualification levels, as this was expected to increase the variation in (the level of) work characteristics and resources (Warr, 1990) of nurses who were working in hospitals, nursing homes, and home care institutions. A total of 4,024 Dutch and 4,354 Polish participants returned the baseline (T0) questionnaire, which means a response rate of 43.2 and 61.4%, respectively. The follow-up questionnaire (T1) was returned by 2,433 (25.1%) and 4,547 (64.5%) nurses, respectively. Table 1 provides some descriptives of the Dutch and Polish sample (distribution of age, gender, and type of institution).

**TABLE 1:** Demographics, descriptives and reliabilities for the samples of the Netherlands and Poland (N ranges from 1,150 to 4,200).

**#**	**Variable**	**Range**	**α**	**M/%**	***SD***	**α**	**M/%**	***SD***
			**The Netherlands**			**Poland**		
	Age	18–64/70	–	38.20	9.73	–	38.72	7.63
	Age in two groups	–	–		–	–		–
	−≤40			57.3			57.9	
	−>40			42.7			42.1	
	Gender	–	–		–	–		–
	- Female			90.7			99.0	
	- Male			9.3			1.0	
	Type of institution	–	–		–	–		–
	- Hospital			62.8%			74.0%	
	- Nursing home			18.7%			4.2%	
	- Home care			18.5%			7.1%	
1	T0 quantitative demands	1–5	0.70	2.99	0.55	0.69	3.38	0.66
2	T0 emotional demands	1–5	0.65	3.45	0.55	0.77	3.49	0.77
3	T0 physical demands	1–4	0.60	2.22	0.70	0.64	2.64	0.80
4	T0 interpersonal relations	1–4*	0.72	2.79	0.49	0.76	2.64	0.58
5	T0 psychological support (1 item)	1–4	–	2.72	0.58	–	2.21	0.80
6	T0 meaning of work	1–5	0.82	4.20	0.58	0.73	3.99	0.82
7	T0 influence at work	1–5	0.71	3.19	0.66	0.83	2.97	0.95
8	T0 opportunities development	1–5	0.70	3.62	0.77	0.76	3.67	0.83
9	T0 burnout	1–5	0.84	1.68	0.60	0.91	2.61	0.99
10	T0 disability**	1–10	0.31 (0.90)	0.46	1.11	0.57 (0.94)	1.46	1.59
11	T0 job satisfaction	1–4	0.70	2.84	0.37	0.78	2.39	0.55
12	T0 institutional commitment	1–5	0.76	3.21	0.66	0.75	3.43	0.88
13	T1 burnout	1–5	0.85	1.65	0.59	0.92	2.80	1.02
14	T1 disability**	1–10	0.34 (0.92)	0.42	1.10	0.47 (0.92)	1.34	1.58
15	T1 job satisfaction	1–4	0.73	2.85	0.40	0.79	2.32	0.56
16	T1 institutional commitment	1–5	0.78	3.05	0.68	0.74	3.25	0.80

*Original range 1–5, recoded into 1–4 (by combining scores 2 and 3) to make variable comparable to T1 measurement. **α between brackets after deletion of Item 1 (number of days kept off work due to Neck Shoulder/Low Backpain complaints). Disability score was calculated following the method described by the author (Von Korff et al., 1992).

### Measures

Three indicators for *working conditions* were included in the present study. *Quantitative demand*s were measured using a four-item scale from the COPSOQ (Copenhagen Psychosocial Questionnaire) (Kristensen and Borritz, 2001), which refers to demands in terms of number of work hours (extensive demand) and/or work pace (intensive demand). An example item was: “How often do you lack time to complete all your work tasks?” Response categories ranged from: 1, “hardly ever,” to 5, “always.” One missing item per subject was allowed for scale calculation. *Emotional demands* were measured using a four-item scale specifically developed for healthcare professionals by De Jonge et al. (1999). Participants were asked to indicate on a five-point rating scale how often they were confronted with “death,” “illness or any other human suffering,” “aggressive patients,” and “troublesome patients” in their work. Response categories ranged from: 1, “never,” to 5, “always.” One missing item per respondent was allowed for scale calculation. *Physical demands* were measured using three items that were constructed by the NEXT study group (Hasselhorn et al., 2003) (physical load major factors in nursing index): (a) “lifting patients in bed without aid,” (b) “maintaining an uncomfortable posture,” and (c) “working in a standing posture.” The response categories for the first two items were: (1) “0 to 1 times a day,” (2) “2–5 times a day,” (3) “6–10 times a day,” and (4) “more than 10 times a day.” The response categories for the third item were: (1) less than 2 h, (2) 2–3 h, (3) 4–5 h, and (4) 6 h or more. The final score has been computed as a sum score divided by three. Physical load was considered to be low when scored from 1 through 2, medium when scored from 2.01 through 2.99, and high when scored from 3 through 4. No missing item was allowed to calculate the mean score.

As regards *labor relations*, two indicators were used. *The quality of interpersonal relations* between nurses and five relevant groups in their working environment (i.e., nursing management, the sister/charge nurse, colleagues, doctors, and administration) was assessed using a five-point scale ranging from: 1, “hostile and tense,” to 5, “friendly and relaxed.” No missing item was allowed for calculating the mean score. The original scale range of 1–5 has been recorded into 1–4 (by combining scores 2 and 3) to make the variables measured at T0 and T1 comparable. *Psychological support* was measured by means of one item (Hasselhorn et al., 2003): “Are you satisfied about the psychological support at work?” The response scale ranged from 1, “very unsatisfied,” to 4, “very satisfied.”

As regards *work content*, we incorporated two indicators. *Meaning of work* was measured using three items from the COPSOQ (Kristensen and Borritz, 2001) (“Is your work meaningful?”, “Do you feel that the work you do is important?”, and “Do you feel motivated and involved in your work?”). The possible scale range was from: 1, “to a very small extent,” to 5, “to a large extent.” No missing items were allowed for calculation of the means. *Influence at work* was measured using a four-item scale containing modified items based on the Demand–Control Questionnaire (Theorell et al., 1988). An example item was: “I can decide for myself how to fulfill the tasks that are assigned to me.” The respondents were asked to indicate on a five-point rating scale how accurate the statements were in relation to their personal occupational situation, with response categories ranging from: 1, “totally inaccurate,” to 5, “totally accurate.” One missing item per participant was allowed for scale construction.

As regards *conditions of employment*, we included one indicator, i.e., opportunities for development. *Opportunities for development* was measured using a four-item scale from the COPSOQ (Kristensen and Borritz, 2001). An example item was: “Do you have the possibility to learn new things through your work?” Response categories ranged from: 1, “to a very small extent,” to 5, “to a large extent.” One missing item per participant was allowed for construction of the scale mean.

As regards the outcomes variables in our research model, the indicators for *health* were burnout and disability. *Burnout* was assessed using a five-item scale taken from the COPSOQ (Kristensen and Borritz, 2001). An example item was: “How often are you emotionally exhausted?” The response scale ranged from: 1, never/almost never, to 5, (almost) every day. One missing item per participant was allowed to calculate the mean score. *Disability* was measured with Von Korff et al.’s (1992) four-item instrument to measure peoples’ pain and/or disability due to low back pain and neck/shoulder pain. An example item was: “Considering the past half year, how much has neck or low back pain interfered with your daily activities?” The response categories ranged from 0 (no interference or change) to 10 (highest interference or very much change). Back- or neck-pain-related disability was considered to be low for nurses scoring 0, medium for nurses scoring from 1 through 2, and high for nurses scoring from 3 through 10. One missing item per participant was allowed for score building. *Job satisfaction* was measured with four items from the COPSOQ (Kristensen and Borritz, 2001). A sample item was: “How pleased are you with your job as a whole?” Responses were made on a four-point rating scale (1, very unsatisfied, to 4, very satisfied). One missing item per participant was allowed to calculate the mean score. *Institutional affective commitment* was measured with Allen and Meyer’s (1996) four-item scale. This scale was used in its original form; however, the wording was – where appropriate – slightly changed. An example item was: “I am proud to belong to this institution.” Responses were made on a five-point rating scale (1, strongly disagree, to 5, strongly agree). One missing item per respondent was allowed to calculate the mean score.

Gender and type of institution (hospital, nursing home, and home care) were included as *control variables* that could be expected to confound relationships between work characteristics and the outcomes. Cronbach’s alphas of all variables are shown in Table 1. The measures used in this study have all proven to be valid and reliable in this study and in previous empirical work.

### Analyses

All data were analyzed for the Netherlands and Poland separately. Prior to testing the hypotheses, we performed dropout analyses (to examine possible mean differences between employees in the panel group and the dropouts) and several preliminary analyses (means, standard deviations, Pearson’s *r* correlations, and Cronbach’s alphas). We first tested whether the proposed pattern of relationships (Hypotheses 1–3) held for the complete Dutch and Polish samples and then tested for differences between younger and older nurses by means of multi-group analyses (MGAs) (Hypotheses 4–7) in IBM AMOS25 (Byrne, 2016).

In all structural equation modeling (SEM) analyses, we simplified the covariance structure by assuming that the latent and observed variables were identical in order to prevent identification problems and unreliable parameter estimates (cf. Schumacker and Lomax, 2010). MGA enables investigating to what extent a proposed pattern of relationships is actually consistent with the observed data in two or more samples simultaneously (Byrne, 2004; Schumacker and Lomax, 2010). Furthermore, with MGA, it is possible to investigate whether a proposed pattern of relationships is invariant (i.e., has the same strength and direction) across different groups (cf. Byrne, 1994, 2016) and whether differences exist between groups (Schumacker and Lomax, 2010).

We used an incomplete panel design to analyze the longitudinal data, using work characteristics measured at T0 and outcome variables measured at both T0 and T1 (e.g., Zapf et al., 1996). We specified the synchronous and cross-lagged paths in accordance with the pattern of relationships as depicted in Figure 1. We allowed the work characteristics as well as the residual errors of the outcome variables to correlate. To assess the overall model fit, several commonly used fit indices were used (cf. Diamantopoulos and Siguaw, 2002; Schumacker and Lomax, 2010; Byrne, 2016): the chi-square statistic, the root mean square residual (RMR), the goodness-of-fit index (GFI), the adjusted goodness-of-fit index (AGFI), the normed fit index (NFI), the comparative fit index (CFI), and the root mean square error of approximation (RMSEA). Models were adjusted based on the modification indices, which provide information as to what specific relationships should be added to the model, when theoretically plausible, in order to improve the fit between the hypothesized model and the data (Byrne, 2016). Nested models were compared by means of the chi-square difference test. Finally, *t*-values were used to assess the significance of specific relationships.

## Results

### Dropout Analyses

In order to rule out selection problems due to panel loss, we determined whether employees in the panel group (who filled out both the T0 and T1 questionnaires) and the dropouts (who only filled out the T0 survey) differed with regard to their mean scores on the research variables and demographics by means of *t*-tests and chi-square difference tests. For most research variables and demographics, the mean differences did not differ significantly between the panel group and the dropouts for both the Netherlands and Poland. More specifically, for the Netherlands though, we found a significant mean difference of 0.82 regarding age. In particular, employees in the panel group [mean (*SD*) = 38.78 (9.70)] were slightly older than the dropouts [Mean (*SD*) = 37.96 (9.74)]; the mean difference was less than one-tenth of the SD. We also found a mean difference of 0.06 regarding burnout, with the dropouts scoring slightly higher [mean (*SD*) = 1.70 (0.55)] in comparison with the panel group [mean (*SD*) = 1.64 (0.55)]; the mean difference was approximately one-tenth of the SD. Finally, the Dutch panel group contained slightly more male nurses (5.6%) than the dropouts (1.9%). For Poland, we found a mean difference of 0.05 regarding quantitative demands, with the panel group scoring slightly higher [mean (*SD*) = 3.41 (0.64)] than the dropouts [mean (*SD*) = 3.56 (0.67)]; the mean difference was less than one-tenth of the SD. With these outcomes, we can conclude that mean differences between the panel group and the dropouts were few and small, and hence that selection problems did not occur in our study.

### Preliminary Analyses

Table 1 shows the demographics of both the Dutch and Polish samples and the means, standard deviations, and internal consistencies of the study variables. Table 2 shows the correlation matrix of all study variables. The reliabilities (in Cronbach’s alphas) of the study variables appeared to be adequate to good. The pattern of correlations was generally in line with Hypotheses 1 and 2 in both samples (the Netherlands and Poland), but opportunities for development tended not to correlate with institutional commitment. In addition, meaning of work was correlated with job satisfaction (as hypothesized) but also with institutional commitment. The variables quality of interpersonal relations and psychological support appeared to be correlated with both burnout (as hypothesized) and job satisfaction.

**TABLE 2:** Correlations between the study variables (Netherlands: left lower corner; Poland: right upper corner; N ranges from 1,150 to 4,200).

		**1**	**2**	**3**	**4**	**5**	**6**	**7**	**8**	**9**	**10**	**11**	**12**	**13**	**14**	**15**	**16**
1	T0 quantitative demands	–	0.29*	0.31*	−0.23*	−0.28*	−0.12*	−0.35*	0.05*	0.27*	0.18*	−0.35*	0.21*	0.16*	0.12*	−0.17*	−0.12*
2	T0 emotional demands	0.72*	–	0.18*	−0.04*	−0.12*	–0.02	−0.10*	0.02	0.18*	0.14*	−0.21*	−0.09*	0.12*	0.11*	−0.11*	–0.04
3	T0 physical demands	0.40*	0.28*	–	−0.07*	−0.13*	–0.01	−0.15*	0.03	0.15*	0.15*	−0.19*	−0.07*	0.06*	0.07*	−0.10*	–0.04
4	T0 interpersonal relations	−0.16*	0.03	−0.06*	–	0.42*	0.27*	0.28*	0.04*	−0.21*	−0.08*	0.34*	0.38*	−0.09*	–0.05	0.18*	0.19*
5	T0 psychological support	−0.24*	−0.11*	−0.18*	0.27*	–	0.23*	0.22*	0.02	−0.26*	−0.15*	0.56*	0.37*	−0.16*	−0.12*	0.21*	0.16*
6	T0 meaning of work	0.04*	0.05*	0.01	0.19*	0.15*	–	0.22*	0.21*	−0.19*	−0.09*	0.31*	0.41*	−0.09*	–0.04	0.17*	0.21*
7	T0 influence at work	−0.36*	−0.08*	−0.21*	0.17*	0.22*	0.14*	–	0.06*	−0.18*	−0.08*	0.25*	0.27*	−0.10*	–0.04	0.15*	0.19*
8	T0 opportunities development	0.03*	0.09*	0.03	0.10*	0.07*	0.28*	0.12*	–	–0.01	0.03	0.06*	0.10*	−0.05*	–0.03	0.08*	0.15*
9	T0 burnout	0.25*	0.12*	0.15*	−0.13*	−0.18*	−0.06*	−0.15*	–0.01	–	0.37*	−0.30*	−0.20*	0.35*	0.17*	−0.15*	−0.10*
10	T0 disability	0.09*	0.05*	0.09*	−0.09*	−0.09*	0.03	0.05*	–0.02	0.23*	–	−0.20*	−0.10*	0.22*	0.34*	−0.13*	−0.05*
11	T0 job satisfaction	−0.30*	−0.15*	−0.23*	0.27*	0.43*	0.28*	0.26*	0.15*	−0.23*	−0.13*	–	0.43*	−0.16*	−0.13*	0.29*	0.19*
12	T0 institutional commitment	−0.13*	−0.10*	−0.15*	0.21*	0.26*	0.26*	0.19*	0.10*	−0.07*	−0.03*	0.36*	–	−0.10*	−0.05*	0.22*	0.34*
13	T1 Burnout	0.18*	0.07*	0.07*	−0.11*	−0.16*	−0.16*	−0.09*	–0.02	0.58*	0.15*	−0.13*	−0.07*	–	0.37*	−0.35*	−0.22*
14	T1 disability	0.10*	0.05	0.07*	–0.04	−0.09*	0.09*	–0.02	0.04	0.17*	0.33*	−0.10*	–0.03	0.21*	–	−0.21*	−0.09*
15	T1 job satisfaction	−0.20*	−0.09*	−0.17*	0.19*	0.25*	0.25*	0.13*	0.02	−0.21*	−0.13*	0.39*	0.18*	−0.26*	−0.14*	–	0.39*
16	T1 institutional commitment	−0.13*	−0.09*	−0.17*	0.19*	0.20*	0.20*	0.09*	0.05	−0.11*	–0.05	0.26*	0.57*	−0.11*	–0.04	0.32*	–

*p < 0.05.

### Testing the Associations Between Work Characteristics and Outcomes

**FIGURE 2:**
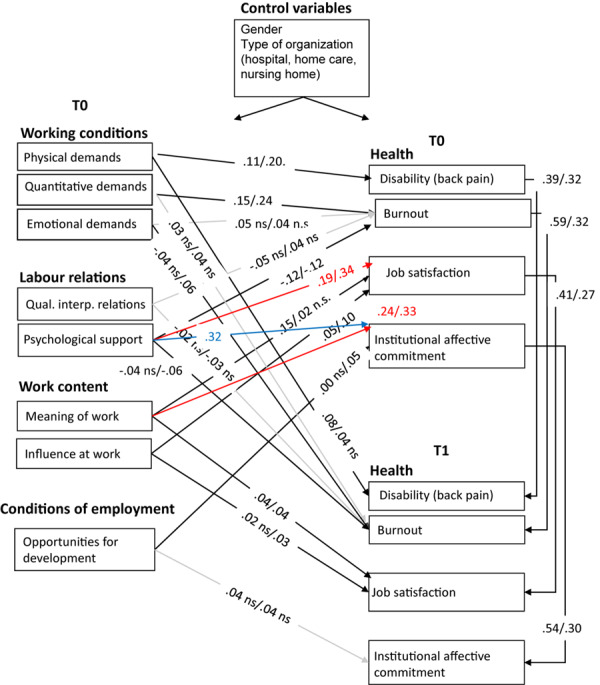
Path coefficients M2 for NL/Poland, all nurses. Red paths have been added for both countries based upon the modification indices; blue path has been added in Poland only based upon modification index; gray paths indicate non-significance for both countries; n.s. implies non-significance for one country; model fit in NL and Poland appears to be good after having added these paths.

In the Dutch sample, the fit of base model M1 was not optimal, so based on modification indices, the following relationships were added (one by one): psychological support to job satisfaction T0 and meaning of work to institutional commitment T0. This adjusted model M2 fitted significantly better (see Table 3). As shown in Figure 2, the hypothesized pattern of relationships was generally supported (Hypotheses 1 and 2 were largely confirmed). In particular, as regards Hypothesis 1 (impact on health), the Dutch nurses’ disability was indeed predicted by physical demands, both in the synchronous and cross-lagged analyses (see Figure 2). The path between psychological support and burnout was significant, yet at T0 only. Contrary to our expectations, the synchronous paths between emotional demands and quality of interpersonal relations, on the one hand, and burnout, on the other hand, were not significant. As regards Hypothesis 2, both the synchronous paths between meaning of work and of influence at work, on the one hand, and job satisfaction were significant. Meaning of work was also positively related to institutional affective commitment T0 (added path) and to job satisfaction at T1 in the Dutch sample. Contrary to our expectations, opportunities for development was unrelated to institutional affective commitment at T0 (with these outcomes, Hypothesis 3 was not confirmed with our data).

**TABLE 3:** Fit measures for all employees and per age group (<40 and >40) for the Netherlands and Poland.

	**χ^2^ (df)**	**Δχ^2^ (df)**	**RMR**	**GFI**	**AGFI**	**NFI**	**CFI**	**RMSEA**
**All employees NL**								
M1 base model	519.493* (68)		0.030	0.948	0.868	0.815	0.830	0.083
M2 adjusted model	333.029* (66)	186.464* (2)	0.025	0.964	0.906	0.88	0.90	0.065
**All employees Poland**								
M1 base model	1000.836* (68)		0.062	0.935	0.835	0.786	0.794	0.101
M2 adjusted model	378.245* (65)	622.591* (3)	0.048	0.971	0.923	0.919	0.931	0.060
**Young nurses NL**								
M1 base model	311.041* (68)		0.026	0.947	0.867	0.806	0.832	0.080
M2 adjusted model	195.097* (66)	115.944* (2)	0.021	0.965	0.908	0.878	0.911	0.059
**Old nurses NL**								
M1 base model	266.856* (68)		0.037	0.937	0.841	0.805	0.836	0.086
M2 adjusted model	157.316* (65)	109.541* (3)	0.031	0.958	0.891	0.885	0.924	0.060
**Young nurses Poland**								
M1 base model	634.397* (68)		0.060	0.932	0.828	0.782	0.794	0.101
M2 adjusted model	268.529* (65)	365.868* (3)	0.049	0.966	0.911	0.908	0.926	0.062
**Old nurses Poland**								
M1 base model	417.311* (68)		0.068	0.928	0.820	0.772	0.792	0.100
M2 adjusted model	189.524* (65)	227.787 *(3)	0.055	0.962	0.901	0.896	0.926	0.061

*p < 0.05.

Considering the cross-lagged paths, the number of significant relationships was relatively low due to the strong stabilities of the outcome variables over time. The cross-lagged path between physical demands and disability was significant for the Dutch nurses. Moreover, meaning of work was related to job satisfaction at T1 as well.

Also, in the Polish sample, the fit of base model M1 was not optimal. Based on modification indices, the following relationships were therefore added (one by one): psychological support to job satisfaction T0, psychological support to institutional commitment T0, and meaning of work to institutional affective commitment at T0. This adjusted model M2 fitted significantly better (see Table 3). As shown in Figure 2, also in this sample, the hypothesized pattern of relationships was generally supported (Hypotheses 1 and 2 were largely confirmed), but several paths had to be added to achieve optimal model fit. The synchronous path between emotional demands and burnout was not significant (just as in the Dutch sample). As regards Hypothesis 2, only influence at work appeared to be related to job satisfaction. In addition, meaning of work had a strong relationship with institutional commitment at T0 and was not significantly related to job satisfaction at T0. Hypothesis 3 was only weakly confirmed when using the synchronous analysis in the Polish sample.

Considering the cross-lagged paths for the Polish sample, emotional demands and psychological support were significantly related to burnout at T1. Moreover, both meaning of work and influence at work appeared to be significantly related to job satisfaction at T1.

### Testing the Impact of Age: Multi-Group Analyses

In accordance with suggestions by Byrne (2004), we tested the hypothesized models in the younger and older groups separately prior to performing MGA. Table 3 shows the model fit of the base models (M1) and the adjusted models (M2) for younger (<40) and older (≥41) Dutch and Polish nurses. For the younger nurses in the Netherlands, we added the following paths: psychological support to job satisfaction T0 and meaning of work to institutional commitment T0. For the older Dutch nurses, we added these paths as well, plus an additional path from psychological support to institutional commitment T0.

For the younger nurses in Poland, we added paths from psychological support, on the one hand, to job satisfaction T0 and institutional commitment T0, on the other hand. In addition, we added a path from meaning of work to institutional affective commitment at T0. For the older nurses in Poland, we added paths from psychological support to job satisfaction T0 and institutional commitment T0 (similar to the younger nurses), and from quality of interpersonal relations to institutional commitment at T0. These adjusted models M2 fitted significantly better than the base models M1 for all four samples and were used as input for the MGA. In these MGAs, we compared a fully constrained invariant model M1 with two less constrained fully and partially non-invariant models M2 and M3 by means of the chi-square difference test (see Table 4).

**TABLE 4:** Fit measures and chi-square difference tests of the nested models in the Multi-Group Analyses (young versus old) for the Netherlands and Poland.

	***Chi-2 (df)***	***Comparison***	***ΔChi-2 (df)***	***NFI***	***CFI***	***RMSEA***
**THE NETHERLANDS**						
M1 fully constrained invariant model^a^	364.583* (150)			0.876	0.917	0.040
M2 partially invariant^b^	326.647* (133)	M1-M2	37.936* (17)	0.890	0.927	0.039
M3 fully non-invariant model^c^	325.523* (130)	M2-M3	1.124 (3)	0.877	0.919	0.039
		M1-M3	39.06* (20)			
**POLAND**						
M1 fully constrained invariant model^a^	442.574* (148)			0.907	0.934	0.039
M2 partially invariant model^d^	414.643* (131)	M1-M2	27.931* (17)	0.912	0.936	0.040
M3 non-invariant model^c^	413.405* (128)	M2-M3	1.238 (3)	0.913	0.936	0.041
		M1-M3	29.142 (20)			

*p < 0.05. ^*a*^All hypothesized and additional paths were specified as invariant for both young and old. ^*b*^All hypothesized and additional paths were set free for both young and old. The path between psychological support and job satisfaction T0 and the paths between opportunities for development and institutional commitment T0 and T1 were specified as invariant. ^*c*^All hypothesized and additional paths were set free for both young and old. ^*d*^All hypothesized and additional paths were set free for both young and old. The paths between psychological support and job satisfaction/institutional commitment T0 and the path between influence at work and job satisfaction T1 specified as invariant.

**FIGURE 3:**
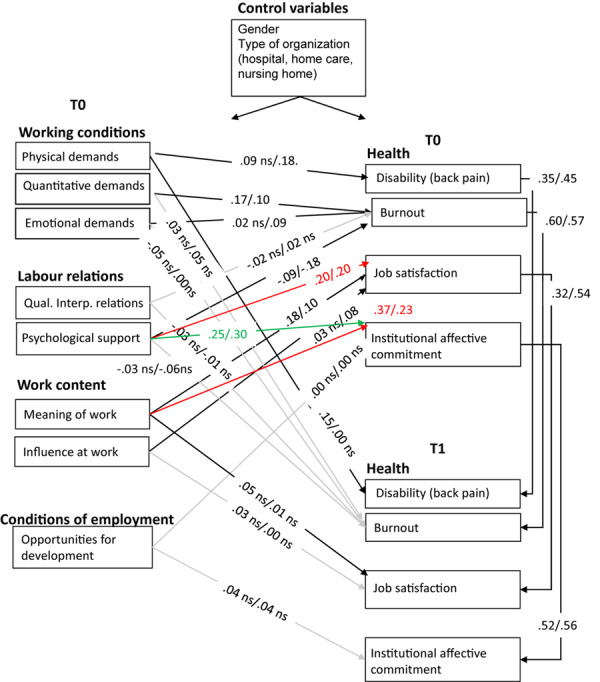
Path coefficients young/old M2 MGA the Netherlands. Red paths were added for both younger and older nurses based on modification indices; green path was added based on modification index in older nurses’ sample; gray paths indicate non-significance for both age groups; n.s. implies non-significance for one age group; model fit for younger and older nurses appears to be good after having added these paths.

For the Dutch sample, we found that M2 and M3 both had a better fit than M1 (invariant model) but that M3 was not better than M2. Hence, M2 (partially invariant) is the best model, and M2 also has the best practical fit indices. This outcome means that the pattern and strength of relations was not similar for younger and older Dutch nurses. Figure 3 shows the path coefficients of younger and older Dutch nurses in M2. This figure shows ample differences between younger and older Dutch nurses. Physical demands relate to disability and emotional demands to burnout in the synchronous analysis for the older but not for the younger nurses (in line with Hypothesis 4). Surprisingly, the cross-lagged path between physical demands and disability is significant for younger nurses but not for the older nurses. Contradictory to Hypothesis 5, the quality of interpersonal relationships was not related to burnout, either for younger or for older nurses. In line with our expectations, psychological support appeared to be more strongly related to burnout for older nurses. Moreover, for both younger and older nurses, psychological support is related to job satisfaction, and meaning of work is related to institutional commitment (additional paths). In addition, for both younger and older nurses, psychological support is related to institutional affective commitment, but more so for older nurses. As regards Hypothesis 6, we found that influence at work was only significantly related to job satisfaction for older nurses but that meaning of work was more relevant for younger nurses. In addition, for both categories of nurses, especially for the younger ones, meaning of work was significantly and strongly related to institutional affective commitment (additional path). Hypothesis 7 could not be confirmed with our data, as opportunities for development did not relate to institutional commitment.

**FIGURE 4:**
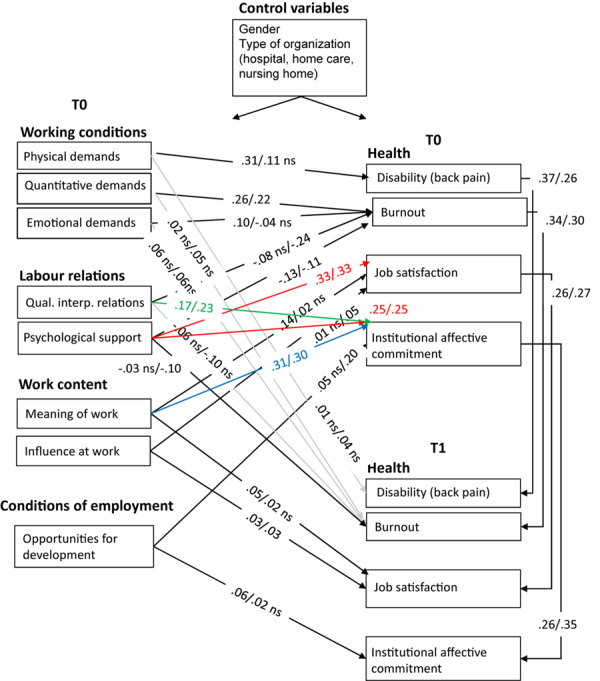
Path coefficients young/old M2 MGA Poland. Red paths were added for both younger and older nurses based on modification indices; blue path was added based on the modification index in younger nurses’ sample; green path was added based on the modification index in older nurses’ sample; gray paths indicate non-significance for both age groups; n.s. implies non-significance for one age group; model fit for younger and older nurses appears to be good after having added these paths.

For the Polish sample, we found that M2 had a better fit than M1 (invariant model), but M3 did not. Hence, M2 (partially invariant) is the best model, and it also appeared to have the best practical fit indices on average (see Table 4). This means that the pattern and strength of relationships is not similar for younger and older Polish nurses (just as for the Dutch nurses). Figure 4 shows the path coefficients of younger and older Polish nurses in M2. Contrary to the findings in the Dutch sample and in contradiction to Hypothesis 4, the relationships between the various types of demands, on the one hand, and disability and burnout, on the other hand, are stronger for the younger Polish nurses than for their older counterparts. In accordance with Hypothesis 5, we found the relationship between quality of interpersonal relationships and burnout to be stronger for older nurses, yet only in the synchronous paths. In case psychological support was the predictor, the cross-lagged paths indicated that older nurses benefit from more psychological support. In accordance with Hypothesis 6 (and the Dutch findings), influence at work was more important in the light of one’s job satisfaction for older nurses synchronously. However, contrary to our expectations, meaning of work appeared to be more relevant for younger nurses in the prediction of job satisfaction (in line with the outcomes for their Dutch counterparts). In addition, for both young and old nurses, meaning of work was significantly and strongly related to institutional affective commitment (additional path). Hypothesis 6 was not confirmed for the cross-lagged paths. As regards Hypothesis 7, the results are a bit ambiguous: The synchronous path between opportunities for development and institutional affective commitment was stronger for older nurses (contrary to our expectations), while the cross-lagged path (over time) was stronger for younger nurses. For both younger and older Polish nurses, we found that the labor relations variables, that is, quality of interpersonal relationships and psychological support, were related to institutional commitment. So, for the Polish nurses, meaning of work and labor relations were more important in the light of institutional affective commitment than opportunities for development. Table 5 provides a complete overview of the extent to which our hypotheses have been confirmed and the relationships that have been added.

**TABLE 5:** Overview of confirmation of hypotheses tested (cross-sectional; cl = cross-lagged confirmation as well; ns = not significant).

**Hypothesis**		**NL**	**POL**	**Added relationships (not hypothesized)**
1	*Health is predicted by working conditions and labor relations:*	*Largely confirmed*	*Largely confirmed*	–
	- Quantitative demands = > burnout	Confirmed	Confirmed	
	- Emotional demands = > burnout	Not confirmed (ns)	Only cl	
	- Physical demands = > disability	Confirmed (cl)	Confirmed	
	- Quality of interpersonal relations = > burnout	Not confirmed (ns)	Not confirmed (ns)	
	- Psychological support = > burnout	Confirmed	Confirmed (cl)	
2	*Job satisfaction is predicted by work content:*	*Confirmed*	*Largely confirmed*	Psychological support (NL and POL)
	- Meaning of work = > satisfaction	Confirmed (cl)	Only cl	
	- Influence at work = > satisfaction	Confirmed	Confirmed (cl)	
3	*Institutional affective commitment is predicted by conditions of employment:*	*Not confirmed*	*Confirmed*	Psychological support (POL)
	- Opportunities for development = > commitment	Not confirmed (ns)	Confirmed	Meaning of work (NL and POL)
4	*Age impacts the relationship between working conditions and health such that the positive relationship between quantitative and emotional demands and nurses’ burnout, and the positive relationship between physical demands and nurses’ disability are stronger for older compared to younger workers:*	*Largely confirmed*	*Not confirmed*	–
	- Quantitative demands = > burnout	Not confirmed (relationship is stronger for younger nurses)	Not confirmed (relationship is stronger for younger nurses)	
	- Emotional demands = > burnout	Confirmed	Not confirmed (relationship is ns for older nurses)	
	- Physical demands = > disability	Confirmed (not cl)	Not confirmed (relationship is stronger for younger nurses)	
5	*Age impacts the relationship between labor relations and health such that the positive relationship between the quality of interpersonal relations and psychological support, on the one hand, and nurses’ burnout on the other hand, is stronger for older compared to younger workers:*	*Partially confirmed*	*Partially confirmed*	–
	- Quality of interpersonal relations = > burnout	Not confirmed (relationship is ns for both young and old)	Confirmed	
	- Psychological support = > burnout	Confirmed	Not confirmed (relationship is somewhat stronger for younger nurses)	
6	*Age impacts the relationship between work content and job satisfaction such that the positive relationship between meaning of work and influence at work with nurses’ job satisfaction is stronger for older compared to younger workers:* - Meaning of work = > satisfaction - Influence at work = > satisfaction	*Partially confirmed* Not confirmed (relationship is stronger for younger nurses) Confirmed	*Partially confirmed* Not confirmed (relationship is stronger for younger nurses) Confirmed	Psychological support (NL and POL): - Equally strong for young and old
7	*Age impacts the relationship between conditions of employment and institutional affective commitment such that the positive relationship between opportunities for development and institutional affective commitment is weaker for older workers:*	*Not confirmed*	*Not confirmed*	Psychological support (NL and POL): - NL: stronger for old - POL: equally strong for young and old
	- Opportunities for development = > commitment	Not confirmed (relationship is ns for both young and old)	Not confirmed (relationship is stronger for older nurses)	Quality of interpersonal relations (POL): - Stronger for old Meaning of work (NL and POL): - stronger for young

## Discussion

### Reflection Upon the Results

As the nursing profession comprises a highly demanding field, while at the same time suffering from a severe shortage of workers, this study focused on the impact of work characteristics and nurses’ experiences that were supposed to be of essential importance to maintain the sustainable employability of nurses throughout the life span. Four categories of work characteristics were taken into account – (1) working conditions, (2) labor relations, (3) work content, and (4) conditions of employment – and we examined their associations with nurses’ health, satisfaction, and commitment.

From a theoretical point of view, our study builds on and elaborates the model of Houkes et al. (2001). Despite being coined almost 20 years ago, we contend that this model has yet to receive the empirical attention it deserves. In contrast to popular models, such as the model for Work, Stress, and Health (Kompier and Marcelissen, 1990; Kompier and Di Martino, 1995), the DCS model (Johnson and Hall, 1988; Karasek and Theorell, 1990), and the ERI model (Siegrist, 1996), the model of Houkes et al. (2001) allows for a further differentiation of demanding and resourceful job characteristics and for studying their unique impact on various aspects of employee functioning. Our results confirm the earlier findings of Houkes et al. (2001, 2003) in different samples and confirm that more accurate predictions regarding the relationships between a relevant range of work characteristics and outcome variables are possible. This allows for the refinement of well-known job design models and more concrete practical recommendations tailored to the problems at hand, as will be discussed below.

In addition, building upon SOC theory and SST, we investigated whether the pattern of relationships is different for younger (<40 years) versus older (≥40 years) nurses. As in previous scholarly work (see, for instance, Kuokkanen et al., 2003; Vahey et al., 2004), age is often included as a covariate or confounder. Our findings show that such practices may mask important differences in the relationships between work characteristics and outcomes for different age groups and, hence, may limit our theoretical understanding of the impact of work across the life span and the practical recommendations that flow from it. An important contribution of this work is thus that it moves the empirical research on aging at work forward and sheds further light on how age should be taken into account in the job design literature (Schreurs et al., 2012).

A third contribution of this research is that we add to the domain of comparative career research (Thomas and Inkson, 2007), by investigating nurses who are working in Dutch and Polish healthcare institutions. Given the differences in outcomes between the two countries, we argue that the influence of economic, legal, and political characteristics of a society, in relation to the nursing field, should not be ignored by important stakeholders, as they do have an effect on attitudes, beliefs, perceptions, and expectations that people have about work characteristics and their outcomes (Thomas and Inkson, 200).

From our empirical model testing, we may conclude that for both the Dutch and the Polish nurses, our proposed pattern of relationships between work characteristics and outcome variables was generally supported, although we had to add several paths to achieve a better model fit for both countries’ samples. These study results are in line with previous empirical studies among nurses that focused on one of the three outcome variables (e.g., Estryn-Behar et al., 2007; Toode et al., 2011; Van der Heijden et al., 2017; Li et al., 2018; Lu et al., 2019).

More specifically, as regards Hypothesis 1 and in line with Houkes et al. (2001), we found health (burnout) to be primarily predicted by working conditions (i.e., quantitative job demands) and labor relations (i.e., psychological support). Emotional demands [not included in the studies by Houkes et al. (2001)] appeared to be unrelated to burnout among both Dutch and Polish nurses. It might be that this professional group is well able to deal with the emotional demands that are an integral and probably anticipated part of their work. The significant relationship between physical demands and disability (not studied by Houkes and colleagues) shows – in line with the Demand-Induced Strain Compensation (DISC) model by De Jonge et al. (2008) – that particular types of job demands relate to matching health outcomes, which may be an important addition to the model of Houkes et al. (2001).

Pertaining to Hypothesis 2, job satisfaction appeared to be related with work content (i.e., influence at work). The relationship between the other indicator of work content (i.e., meaning of work) appeared to be less relevant for nurses’ job satisfaction in both countries. This may be due to the relatively high mean score and low variance on meaning of work in both countries, leading to ceiling effects: Meaning of work may be so salient for this professional group that a further increase in meaning of work does not lead to an increase in job satisfaction. Next to work content, labor relations (i.e., psychological support) also appeared to be an important predictor of job satisfaction in the current study. Job satisfaction is a broader motivational outcome than mere intrinsic motivation, which was the focus in the study of Houkes et al. (2001), which may explain why this additional relationship was found.

Finally, as regards Hypothesis 3, the relationship between conditions of employment (i.e., opportunities for development) and institutional affective commitment was significant for the Polish nurses but not for the Dutch ones. Interestingly, however, for both the Netherlands and Poland, we found meaning of work to be related to institutional affective commitment, and this relationship was even stronger than the relationship between conditions of employment and institutional affective commitment. Apparently, nurses may be committed to their work because they can perform meaningful work, and less so because of personal development goals. This stresses the importance of this factor in the light of protecting nurses from prematurely leaving the organization or even their profession as a whole.

Comparing the younger and older nurses (Hypotheses 4–7), we found ample support for our assumption that the hypothesized relationships might be impacted by age. In particular, age seemed to moderate the relationship between working conditions and health (Hypothesis 4), yet results were different in the Dutch compared to the Polish sample. In particular, in the Netherlands, physical demands are associated with older nurses’ disability (synchronous analysis). Yet, as regards the analysis over time, it appears that only for the younger nurses, the higher the amount of physical demands, the higher their disability. Emotional demands go together with more burnout for the older nurses (synchronous analysis). Surprisingly, younger nurses experienced more health issues due to poor working conditions compared to older workers in Poland, but emotional and physical demands were more problematic for older workers in the Netherlands, although the latter could not be confirmed when considering the analyses over time. Such findings could be in line with theories on aging, but it is not easy to explain why these relationships differ across countries and were not stable across cross-sectional and longitudinal analysis. We therefore suggest taking into account multi-wave approaches to better disentangle these findings.

As regards the impact of age on the relationship between labor relations and health (Hypothesis 5), we found support for a stronger negative association between quality of interpersonal relationships and burnout for older nurses, yet only in Poland. For the Dutch nurses, psychological support appeared to prevent burnout and, in line with our expectations, indeed more so for the older ones.

We also found some support that age impacts on the relationship between work content and job satisfaction (Hypothesis 6). Across samples, influence at work was more strongly related to job satisfaction for older than for younger nurses. This indicates that older employees benefit relatively more from having a voice in the work context, while this is less relevant for younger workers. As regards meaning of work, it appeared that, in particular for younger nurses, this entails more job satisfaction.

Overall, across the Dutch and Polish samples, we found no impact of age on the relationship between conditions of employment and commitment in the hypothesized direction (Hypothesis 7).

### Limitations of the Study and Recommendations for Future Research

This study has some limitations. First, all data were collected using surveys, herewith opening the possibility of response set consistencies. Moreover, as we used self-report measures for both the predictor variables and the outcomes, a common-method bias may exist (Doty and Glick, 1998; Podsakoff et al., 2003). Another limitation comprises the possibility of chance capitalization, given the large amount of relationships tested. However, we have tried our utmost to prevent this by testing the model relationships simultaneously. Future research wherein data on both nurses’ self-assessments and supervisors’ assessments are gathered to compare their perceptions on the work characteristics might be interesting. In addition, as we focused specifically on nurses, our empirical findings are highly relevant for this particular professional group. Also, to increase generalizability across healthcare settings, nurses were sampled across hospitals, nursing homes, and home care institutions. Future research might be aimed at cross-validation at different professional settings and/or to other countries. In addition, personality characteristics may moderate the effect of work characteristics on health, job satisfaction, and organizational commitment. More scholarly work is needed, for instance, using the “Big Five” (Costa and McCrae, 1992) in order to gain more insight into possible moderating effects. It is also important to continue empirical work in this field to better understand the possible impact of age, using different cutoff points, such as 50 or 55 years of age [cf. Armstrong-Stassen and Schlosser (2008) who found that job development climate played an important role in the retention of workers older than 50 years old]. We also recommend future research incorporating different age conceptualizations to shed more light on the possible role of age [see, for instance, the categorization by Sterns and Doverspike (1989) into chronological age, functional or performance-based age, psychosocial or subjective age, organizational age, and the concept of life span age]. Other factors that may have predictive validity in the light of nurses’ health, satisfaction, and commitment, such as leadership style, work–family interference, rostering, shift work, positive and negative affectivity, effort–reward imbalance, to mention but a few appealing ones, should be taken into account in future empirical work as well.

Last but not least, we recommend expanding the impact of work characteristics on nurses’ health, satisfaction, and commitment to patient-related outcome variables such as medical errors and patient experiences and satisfaction. It was the primary aim of this study to get more insight into specific determinants of employee well-being, but in the context of healthcare, it seems relevant to study the impact of these work characteristics on patient satisfaction as well.

### Implications for Practice

This study has increased our insights into the impact of work characteristics on key outcomes in the nursing profession. All in all, this empirical work indicates that managers in healthcare institutions have to pay careful attention to the working conditions, labor relations, and employment conditions in order to protect nurses’ health, motivation, and career decisions. First, our results point out that depending on the most prevalent problems in their particular organization (health issues, motivational problems, or commitment issues), managers could focus on particular work characteristics. In general, our results point out that in the case of burnout, managers should primarily focus on lowering quantitative demands, for example, through preventing too-high levels of demands that are increasing nurses’ workload, by reorganizing work, through job crafting (Parker et al., 2017b), or by increasing psychological support, for example, through encouraging and supporting fruitful interpersonal relationships on the work floor (Jungert et al., 2018). When satisfaction is low, the focus should furthermore be on increasing employee influence, for example, by enabling nurses to participate in decision-making and governance arrangements (Uchiyama et al., 2013). In general, the results thus indicate that managers have to protect their nurses from a too-high amount of quantitative, emotional, and physical demands and have to ensure that there is enough room for nurses’ influence at the workplace. This study also stresses the importance of meaningful work, not only in the light of the added value for nurses’ job satisfaction but, in particular, given its unexpected impact in the light of nurses’ commitment. Managers in healthcare institutions can furthermore increase nurses’ job satisfaction by providing ample psychological support. Psychological support is found to be important in the light of combatting burnout symptoms but also increases one’s job satisfaction. Our findings support the importance of one’s direct supervisor in the light of sustainable career theory (Van der Heijden and De Vos, 2015). Overall, nurses seem to have chosen their profession mainly because they want to do meaningful work and less so because of the opportunities for further development. Therefore, we argue that managers in healthcare organizations who want to foster passion at work and who supervise nurses for whom emotional demands and helping other people can even be a challenge instead of a burden (Bakker and Sanz-Vergel, 2013) should offer employees meaningful work and promote work valuation (Vallerand and Houlfort, 2003).

Second, apart from this generic advise, our results also clearly point to the importance of generating more in-depth understanding of the relationships between work characteristics and nurses’ health, job satisfaction, and commitment in one’s particular context, both in terms of the age of the staff and country. This implies that within each context, managers should allow for a thorough assessment of the work characteristics and functioning of their staff and the relations between them before considering particular interventions. Hence, we stress the importance of a non-normative, tailor-made approach to aging at work, herewith doing justice to idiosyncrasy (Van der Heijden and De Vos, 2015) in order to protect and further the career sustainability of all workers across the life span.

## Conclusion

To conclude, the results of this study can be translated into further clear recommendations for management in healthcare settings. Management should invest in the attractiveness of the profession and the quality of the practice environment aimed at the inclusion and retention of nurses across the life span. To improve nurses’ health, satisfaction, and commitment, they should pay attention to the working conditions, labor relations, work content, and employment conditions.

## Data Availability Statement

The datasets generated for this study are available on request to the corresponding author.

## Ethics Statement

The studies involving human participants were reviewed and approved by Ethics committee, University of Wuppertal. The patients/participants provided their written informed consent to participate in this study.

## Author Contributions

BV, IH, and AV worked on design, modeling and analyses, and writing. KC worked on writing. All authors listed have made a substantial, direct and intellectual contribution to the work, and approved it for publication.

## Conflict of Interest

The authors declare that the research was conducted in the absence of any commercial or financial relationships that could be construed as a potential conflict of interest.

## Footnotes

1 The NEXT study was financed by the European Commission within the Fifth Framework, Project ID: QLK-6-CT-2001-00475.
